# Pairwise shared genomic segment analysis in three Utah high-risk breast cancer pedigrees

**DOI:** 10.1186/1471-2164-13-676

**Published:** 2012-11-28

**Authors:** Zheng Cai, Alun Thomas, Craig Teerlink, James M Farnham, Lisa A Cannon-Albright, Nicola J Camp

**Affiliations:** 1 Department of Biomedical Informatics, University of Utah Medical School, Health Sciences Education Building, Salt Lake City, UT, 84112, USA; 2 Division of Genetic Epidemiology, University of Utah Medical School, 391 Chipeta Way Suite D, Salt Lake City, UT, 84108, USA; 3 George E. Wahlen Department of Veterans Affairs Medical Center, 500 Foothill Drive, Salt Lake City, UT, 84148, USA

**Keywords:** Breast cancer, High-risk pedigrees, Susceptibility, Germline, Genomic sharing

## Abstract

**Background:**

We applied a new weighted pairwise shared genomic segment (pSGS) analysis for susceptibility gene localization to high-density genomewide SNP data in three extended high-risk breast cancer pedigrees.

**Results:**

Using this method, four genomewide suggestive regions were identified on chromosomes 2, 4, 7 and 8, and a borderline suggestive region on chromosome 14. Seven additional regions with at least nominal evidence were observed. Of particular note among these total twelve regions were three regions that were identified in two pedigrees each; chromosomes 4, 7 and 14. Follow-up two-pedigree pSGS analyses further indicated excessive genomic sharing across the pedigrees in all three regions, suggesting that the underlying susceptibility alleles in those regions may be shared in common. In general, the pSGS regions identified were quite large (average 32.2 Mb), however, the range was wide (0.3 – 88.2 Mb). Several of the regions identified overlapped with loci and genes that have been previously implicated in breast cancer risk, including *NBS1*, *BRCA1* and *RAD51L1*.

**Conclusions:**

Our analyses have provided several loci of interest to pursue in these high-risk pedigrees and illustrate the utility of the weighted pSGS method and extended pedigrees for gene mapping in complex diseases. A focused sequencing effort across these loci in the sharing individuals is the natural next step to further map the critical underlying susceptibility variants in these regions.

## Background

Breast cancer (MIM #114480) is the most prevalent cancer among women in developed countries [1]. It is a common, complex disease, including substantial genetic heterogeneity with respect to both loci and alleles. To date, many germ-line variants in multiple genes have been confirmed to increase risk for breast cancer [2]. However, the majority of hereditary breast cancer remains unexplained and there are clearly more risk variants to identify. In particular, rare variants are likely to be a part of the missing heritability [3]. Pedigrees selected for excess disease (i.e. high-risk pedigrees) offer the potential for increased genetic homogeneity and enrichment for rare and more penetrant variants. Hence the high-risk pedigree design is advantageous for the detection of rare risk variants. However, although the complexity is arguably reduced, genetic heterogeneity may still remain and can pose a substantial challenge for conventional pedigree-based methods, such as linkage analysis. High-density single nucleotide polymorphism (SNP) data also provide challenges for conventional multi-point pedigree methods because of linkage disequilibrium (LD) between markers and because subtle non-Mendelian genotype errors or inaccuracies of physical position can confuse estimation of the inheritance vectors. Genomewide association is well-suited to high-density SNP arrays, however, the power for this approach lies with the existence of high LD between a SNP on the platform and the underlying risk variant; which is vastly reduced with rare risk variants leading to low power [4,5]. Identity-by-descent (IBD) mapping, such as shared genomic segment (SGS) analysis, in extended pedigrees have been developed precisely for use with high-density SNP platforms and have been suggested to be more powerful than association analysis and traditional linkage analysis for the identification of rare variants [3,6,7]. The probability of IBD is a challenge to calculate in large pedigrees. Conversely, identity-by-state (IBS) is easy to compute. Our SGS methods use excessive lengths of IBS to find regions of IBD. These IBS regions are assessed for significance empirically, conditional on a model for LD and a genetic model (for recombination in the pedigree). Our original SGS method [8] was designed to identify regions of excessive lengths of sharing across all, or all but 1 or 2, cases in a pedigree, which is powerful when the cases within pedigrees are reasonably genetically homogeneous [6]. For common diseases, large high-risk pedigrees may suffer from intra-familial heterogeneity, such as when more than one genetic locus segregates within the same family. In these situations, even at a true risk locus, a greater proportion of the cases may be non-sharers. Recently, we proposed an alternate weighted pairwise SGS (pSGS) method, which combines the sharing evidence across all possible pairs, which in simulated data indicated substantial increased robustness to intra-familial genetic heterogeneity and therefore is likely more useful for mapping common diseases [9].

We performed genomewide pSGS analysis in three Utah high-risk breast cancer pedigrees selected as unlikely to be due to *BRCA1* or *BRCA2*. Regions of excessive sharing in the cases of these pedigrees have good potential for harboring breast cancer susceptibility variants.

## Methods

### High-risk breast cancer pedigrees

Using existing mutation screening and microsatellite linkage data, pedigrees were selected to have low probability of being due to mutations in the genes *BRCA1* and *BRCA2*. Each met the following criteria [10]: (1) the pedigree did not contain any cases known to carry *BRCA1* or *BRCA2* mutations, and (2) the pedigree had no significant linkage to the *BRCA1* or *BRCA2* regions. Hence, a-priori these pedigrees have a low probability of segregating mutations in *BRCA1* or *BRCA2*.

**Figure 1:**
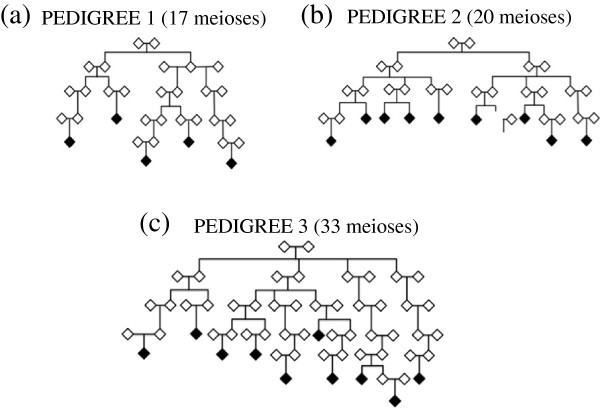
Three extended high-risk breast cancer pedigrees.

The three extended, high-risk Utah pedigrees studied are shown in Figure 1. All pedigrees were descended from European founders. There are no known genealogical links between the pedigrees, as determined by the Utah Population Database (UPDB [11]) which contains up to eleven generations of genealogy. Pedigree 1 contains five cases connected by a total of 17 meioses. Pedigree 2 contains 9 cases connected by 20 meioses. Pedigree 3 consists of 10 cases connected by 33 meioses. Confirmation of cancer diagnoses was obtained from the Utah Cancer Registry (UCR). All other individuals were considered “unknown”, and were not genotyped in this study. These pedigrees are defined as high risk because they contain significantly more female breast cancer than expected using cancer rates calculated from the UPDB (see [12]).

Informed consent was obtained from all participants in this study. This study is approved by the Institutional Review Board at University of Utah.

### Control Samples for estimation of LD

**Figure 2:**
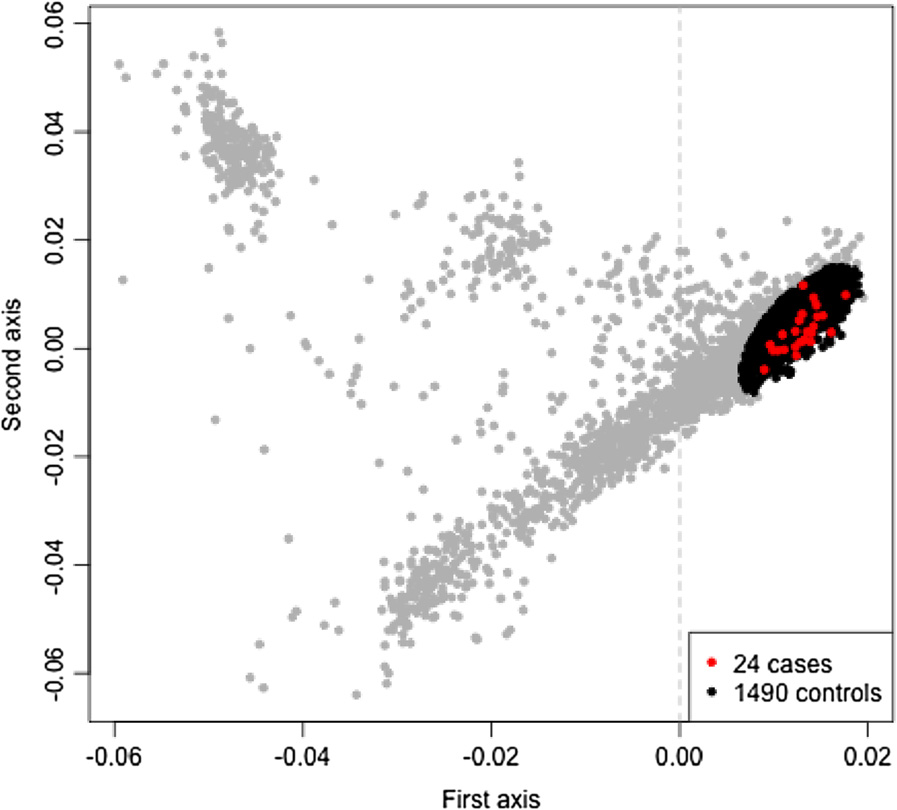
**Principal Component Analysis for selection of iControlDB individuals.** The first and second principal components The 24 breast cancer cases from the three pedigrees are shown in red, and the selected iControlDB individuals are shown in black.

In SGS approaches, control samples are required to estimate genomewide LD structure that is used in the empirical assessment of significance. The primary set of controls used was ascertained locally via the UPDB resource (also the source of the pedigrees). These control individuals were known to be cancer-free and were self-declared Caucasian. These 224 ‘local controls’ comprised 117 males and 107 females. To ensure robustness of our findings, for regions of interest identified from the genomewide pSGS analysis based on the LD model estimated from the local controls, we also assessed significance based on a set of genomically-matched controls. This second set of controls comprised individuals selected from the Illumina Genotype Control Database (iControlDB) (http://www.illumina.com). Principal components analysis was carried out on the set of all self-declared Caucasian samples in iControlDB with genotype data available for the 550K Illumina SNP array. We pruned the 550K Illumina SNPs to a set with r^2^<0.5 and used *smartpca*[13] to extract the first two principal components and identified 1,490 iControlDB individuals who resided within 3 standard deviations of the centroid based on a bivariate normal distribution estimated from the cases. These 1,490 genomically matched controls comprised 949 female and 541 males. Figure 2 illustrates the 1,490 iControlDB individuals selected.

### Genotypes

SNPs from the Illumina 550K array were used. SNPs with a significantly different missing data rate between cases and controls (*p* < 10^-5^), those with a missing rate greater than 5%, a minor allele frequency (MAF) of less than 1%, or significantly different from Hardy-Weinberg Equilibrium (*p* < 10^-4^) were removed. This resulted in a total of 516,475 SNPs genomewide included in the SGS analyses.

### Data analysis

Our primary analysis was a genomewide pSGS analysis. For regions of interest identified by pSGS with at least nominal evidence (p≤0.05) we also performed SGS and multipoint linkage analysis as secondary analyses, for comparison.

#### Shared genomic segment analysis

Thomas et al. [8] proposed a method of SGS analysis based on sharing among all cases in high-risk pedigrees. Assuming biallelic SNP loci with alleles 1 and 2, the three possible genotypes at each locus are 11, 12 and 22. Sharing is impossible between individuals with the two opposite homozygote types (11 and 22), otherwise IBS sharing exists. Therefore, the number of individuals sharing at a locus can be easily calculated on inspection of the number of homozygote individuals at each locus. We define *S*_*i*_ to be the number of cases sharing at least one allele IBS at SNP *i*.

$$S_{i}=N-\min\left(N_{11}^{i}N_{22}^{i}\right)$$

where *N* is the total number of cases in a pedigree, and *N*_11_^*i*^, *N*_22_^*i*^ are the counts of cases homozygous 11 and 22, respectively. Missing genotypes are treated as heterozygotes.

We use *R*_*i*_ (*t*) to indicate the number of consecutive SNPs (which includes the *i*th SNP) with IBS sharing among at least *t* cases (also referred to as a “run length”), where *t* is usually the total number of individuals whose genotypes are in comparison (*t = N*). We recently introduced a new SGS test statistic, the weighted mean pairwise Shared Genomic Segment (pSGS) statistic [9]. It combines evidence from sharing in pairs of cases, weighted by their genetic distance and hence is less influenced by and has improved robustness to intra-familial heterogeneity. Consider a pedigree with *N* cases, and denote *d*_*jk*_ as the number of meioses between cases *j* and *k*, and *R*_*i*_^*jk*^(2) as the run length shared by the pair of cases *j,k* at locus *i*. The test statistic for the pSGS is defined as follows:

$$pSGS_{i}=\frac{1}{\left(\begin{array}{c} N\\ 2 \end{array}\right)\sum_{j=1}^{N-1}\sum_{k=j+1}^{N}d_{\mathrm{jk}}}\overset{N-1}{\underset{j=1}{\sum }}\overset{N}{\underset{k=j+1}{\sum }}d_{\mathrm{jk}}R_{i}^{\mathrm{jk}}\left(2\right)$$

The significance is assessed empirically based on expected sharing under a model that includes LD as described in Thomas [14]. Our methodology is implemented in freely available java software (http://balance.med.utah.edu/wiki/index.php/Access_programs_by_name).

#### pSGS and SGS: LD model

We used FitGMLD to obtain a LD model based on the 224 local control samples using default parameters [15]. This program applies graphical models to estimate a general finite multivariate distribution for allelic association between genetic loci in each autosomal chromosome. In the model, the variables are alleles at each SNP loci, which are indicated using nodes. Edges connect loci that are in LD with each other and SNPs in a chromosome are modeled using a Markov graph. The program iteratively performs phase imputation and estimation of LD model from genotype dataset of unrelated individuals. The method incorporates an error model for genotyping. The program takes computation time in the magnitude of *O*(*nm*), given *n* individuals with *m* genotyped markers [15].

#### pSGS and SGS: Significance assessment

We estimated nominal *p*-values for each locus using Monte Carlo procedures, by comparing the observed lengths to expected lengths under the null. Sharing under the null was achieved using a gene-dropping procedure assuming random mating, Mendelian inheritance and a genetic map for recombination. Founder haplotypes in the pedigree were generated using the estimated LD model. These were segregated through the known pedigree structure using random Mendelian inheritance to generate genotypes for each descendant in the pedigree. Recombinant events were based on an established genetic map [14]. Simulated genotypes were only retained for the studied cases in each pedigree and SGS statistics were calculated using the null data configurations to generate a distribution of lengths shared under the null for each pedigree.

The simulation procedure was implemented using a parallel Java program to improve computational efficiency.

#### pSGS and SGS: Genomewide thresholds

Genomewide thresholds provide a correction for the multiplicity of tests performed across the genome. For SGS methods, the multiple testing corresponds to the number of SGS segments across the genome, and this depends on the pedigree structure (number of meioses between the studied cases) and the sharing statistic considered (pSGS or SGS). Hence, we estimated genomewide thresholds empirically for each pedigree for both pSGS and SGS. A genomewide significant threshold was defined as the level of significance that would be achieved at a rate of 0.05 times per genome under the null (false positive rate per genome, μ=0.05). A genomewide suggestive threshold was defined as the level of significance achieved at a rate of 1 per genome under the null (μ=1.0). To estimate these thresholds we generated 1,000 null genome configurations for each pedigree (matched to the real genetic data for LD and recombination model), performed SGS and pSGS, identified the shared segments and their respective p-values (with p-values estimated based on an empirical distribution of up to 1,000,000 null values). For each pedigree and statistic, the p-values for all segments across all 1,000 genomes were ranked. We identified the 50^th^ ranked p-value across all 1,000 genomes (50/1,000 = 0.05 per genome) to determine the level for the significant threshold; and the 1,000^th^ ranked p-value (1,000/1,000 = 1 per genome) to determine the suggestive threshold.

#### Linkage analysis

We also performed multipoint linkage analysis on each pedigree. In order to eliminate inflation of linkage statistics due to LD, a pruned set of SNPs (n=26,177) were used for the linkage analysis. This set of “LD-free” SNPs had a minimum spacing of 0.1 cM, a minimum heterozygosity of 0.3 (to maintain good information content), and a maximum r^2^ of 0.16 over a sliding 500 kb window in the public available HapMap CEU data, and exceeded an individual call rate of 98% of genotyped subjects. We used an established genetic map [16], plus linearly interpolated SNPs from Human Genome Build 35.1. Allele frequencies were estimated from all genotyped individuals at each SNP. The multipoint linkage analysis was performed using MCLINK, a multipoint Markov chain Monte Carlo (MCMC) linkage method that can analyze extended pedigrees [17]. A cases-only parametric analysis was performed based on a general dominant model.

## Results

For all three pedigrees, nominal evidence was considered to be p≤0.05. For pedigree 1, the empirical genomewide suggestive and significant thresholds for pSGS were p=6.5×10^-3^ and p=3.0×10^-4^, respectively, and the genomewide suggestive and significant thresholds for SGS were p=1.3×10^-4^ and p<1.0×10^-6^. For pedigree 2, no results surpassed the nominal threshold therefore empirical genomewide thresholds were not determined. For pedigree 3, the empirical genomewide suggestive and significance thresholds for pSGS were p=5.0×10^-3^ and p=2.5×10^-4^, respectively, and the suggestive and significant thresholds for SGS were estimated as p=3.8×10^-5^ and p<1.0×10^-6^. Genomewide thresholds for suggestive and significant linkage signals have been previously established to be LODs of 1.86 (p=1.7×10^-3^) and 3.30 (p=4.9×10^-5^), respectively [18].

**Figure 3:**
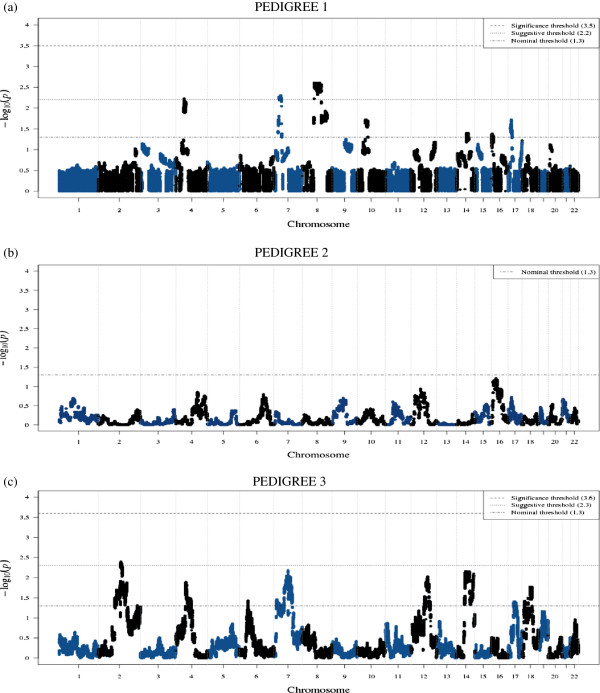
**Genomewide pSGS results.** For pedigree 1, genomewide suggestive and significance pSGS thresholds were estimated to be p=6.5×10^-3^ (−log_10_p=2.2) and p=3.0×10^-4^ (−log_10_p=3.5), respectively. For pedigree 3, genomewide suggestive and significance pSGS thresholds were estimated to be p=5.0×10^-3^ (−log_10_p=2.3) and p=2.5×10^-4^ (−log_10_p=3.6), respectively.

Figure 3 shows the genomewide pSGS results for each pedigree based on a LD model estimated from the local controls. Table 1 illustrates all pSGS regions containing at least nominal evidence. For each of these regions, Table 1 also summarizes the best SGS p-value in the region (all *N* cases sharing) and the multipoint LOD score from linkage analysis. Table 2 shows a comparison between the pSGS p-values attained based on the local controls LD model and those based on the iControlDB individuals LD model and indicates that our results are extremely robust.

**Table 1:** Regions with at least nominal evidence in pSGS (p≤0.05)

**Pedigree (number cases; min. possible sharing)^†^**	**Chromosome**	**Region^€^**	**Length (Mb)**	**pSGS p-value^¥^**	**Average cases sharing (range)**	**SGS**	**Linkage**
1	4*	**37,542,764 - 54,575,432**	**17.0**	**0.0060**	**4.78 (3–5)**	0.00017	1.19
(n=5; min=3)	7*	**16,704,212 - 31,213,647**	**14.5**	**0.0050**	**4.83 (3–5)**	0.00010	**2.62**
	**8**	**38,344,499 - 122,638,989**	**83.3**	**0.0025**	**4.78 (3–5)**	**0.000018**	1.55
	10	28,738,098 - 49,576,878	20.8	0.019	4.78 (3–5)	0.0020	1.25
	14*	66,272,834 - 77,581,481	11.3	0.040	4.78 (3–5)	0.029	1.19
	16	359,567 - 8,197,462	7.8	0.041	4.76 (3–5)	0.009	1.03
	17	10,784,088 - 16,883,680	6.1	0.019	4.81 (3–5)	0.0015	1.30
3	**2**	**74,758,934 - 162,960,873**	**88.2**	**0.0040**	**9.41 (6–10)**	0.00014	1.66
(n=10; min=5)	4*	47,003,076 - 88,807,556	41.8	0.013	9.44 (5–10)	0.00033	0.89
	6	31,320,810 - 31,628,733	0.3	0.037	9.60 (7–10)	0.0062	0.19
	7*	***11,358,235 - 96,674,424***	***85.3***	***0.0065***	***9.40 (6–10)***	0.00025	1.04
	12	67,987,630 - 101,376,241	33.4	0.0095	9.44 (6–10)	0.00020	1.27
	14*	***56,883,760 - 99,254,712***	***42.4***	***0.0070***	***9.43 (6–10)***	0.00050	0.57
	17	32,760,735 - 51,072,912	18.3	0.039	9.33 (6–10)	0.0014	0.03
	18	8,247,249 - 50,460,551	42.2	0.017	9.43 (6–10)	0.0011	1.32

^†^ For each pedigree the total number of cases in the pedigree, and the minimum possible number of cases that can share (i.e. the number of cases sharing cannot go below this value) are shown.

^**€**^Coordinates are based on GRCh37/hg19.

^**¥**^Significance based on a LD map from 224 local controls.

*Overlapping regions are indicated by an asterisk.

For pedigree 1, genomewide pSGS thresholds are p=6.5×10^-3^ and p=3.0×10^-4^, for suggestive and significance, respectively, and corresponding SGS thresholds are p=1.3×10^-4^ and p<1.0×10^-6^.

For pedigree 3, genomewide pSGS thresholds are p=5.0×10^-3^ and p=2.5×10^-4^, for suggestive and significance, respectively, and corresponding SGS thresholds are p=3.8×10^-5^ and p<1.0×10^-6^.

For linkage, genomewide suggestive and significance LOD thresholds are 1.86 and 3.30, respectively, corresponding to p-values of 1.7×10^-3^ and 4.9×10^-5^.

Genomewide suggestive signals are indicated in **bold**. Borderline genomewide suggestive signals are ***bold and italicized***.

**Table 2:** Comparison of pSGS p-values: local controls and iControlDB individuals

**Pedigree**	**Chromosome**	**Region**	**Length (Mb)**	**Local Controls (n=224)**	**iControlDB (n=1,490)**
				**pSGS**	**pSGS**
1	4*	37,542,764 - 54,575,432	17.0	0.0060	0.015
	7*	16,704,212 - 31,213,647	14.5	0.0050	0.0060
	8	38,344,499 - 122,638,989	83.3	0.0025	0.0013
	10	28,738,098 - 49,576,878	20.8	0.019	0.014
	14*	66,272,834 - 77,581,481	11.3	0.040	0.041
	16	359,567 - 8,197,462	7.8	0.041	0.044
	17	10,784,088 - 16,883,680	6.1	0.019	0.017
3	2	74,758,934 - 162,960,873	88.2	0.0040	0.0033
	4*	47,003,076 - 88,807,556	41.8	0.013	0.013
	6	31,320,810 - 31,628,733	0.3	0.037	0.035
	7*	11,358,235 - 96,674,424	85.3	0.0065	0.0055
	12	67,987,630 - 101,376,241	33.4	0.0095	0.0095
	14*	56,883,760 - 99,254,712	42.4	0.0070	0.0055
	17	32,760,735 - 51,072,912	18.3	0.039	0.032
	18	8,247,249 - 50,460,551	42.2	0.017	0.012

Four genomewide suggestive pSGS regions were identified, with an additional two borderline. One of these regions was also genomewide suggestive in the SGS analysis, and one was genomewide suggestive using a dominant linkage analysis (Table 1). Three of the genomewide suggestive pSGS results were found in pedigree 1 on chromosome 4 (37.5-54.6 Mb; p=0.006; μ=0.98, indicating that a finding this extreme would be expected 0.98 times per genome under the null), chromosome 7 (16.7-31.2 Mb; p=0.005; μ=0.82) and chromosome 8 (38.3-122.6 Mb; p=0.0025; μ=0.48). Hence for pedigree 1, three genomewide suggestive regions were observed, compared to less than 1 expected under the null. One genomewide suggestive region was identified in pedigree 3 on chromosome 2 (74.8-163.0 Mb; p=0.004; μ=0.92), in addition two borderline suggestive findings were also identified on chromosome 7 (11.4-96.7 Mb; p=0.0065; μ=1.08, an overlap with the genomewide suggestive region in pedigree 1) and chromosome 14 (56.9-99.3 Mb; p=0.007; μ=1.25, an overlap with a nominal region in pedigree 1). Hence, for pedigree 3, three regions were found compared to a false positive rate of 1.25 or less per genome (3 observed, 1.25 expected). Even accounting for the multiple testing of analyzing three independent pedigrees, we identified 6 signals (5 distinct chromosomal regions) with μ≤1.25, which is greater than the 3.75 would be expected by chance at this significance level.

For all 15 regions shown in Table 1, the average number of cases sharing across the regions was high (>*N*-1 cases), although the range of the number of cases sharing was wide; generally spanning the total range possible. The size of the shared regions also varied quite widely; for the six regions of interest 14.5 – 88.2 Mb (Table 1). Shared regions were defined as the segment of contiguous loci remaining above nominal statistical evidence. Under the null hypothesis (no disease locus) and the assumption that recombinations at each meiosis occur as independent Poisson processes, the expected length of a shared IBD segment is Exponentially distributed with mean 1/*d* Morgans, where *d* is the number of meiosis separating the individuals. For example, a cousin-pair (*d*=4) will share segments of size 25 cM on average. Under the alternate hypothesis that a disease locus exists, the length follows a Gamma distribution with mean 2/*d.* In the cousin-pair example, the average segment length surrounding a shared disease locus would be 50 cM. In our pedigrees, breast cancer pairs ranged from siblings (*d*=2), to pairs separated by 11 meioses (Figure 1). Importantly, it should be noted that our SGS analysis, by design, identifies IBS segments (a less stringent criteria than IBD), with an aim is to identify excessively long regions that are therefore likely to be IBD. For the above reasons, the region lengths we identify may be longer than expected by chance for the given relationships.

Three regions on chromosomes 4, 7 and 14 showed overlapping evidence in pedigrees 1 and 3 (Table 1). To investigate these three regions for evidence of common sharing across pedigrees, we performed two-pedigree pSGS analyses across all cases in pedigrees 1 and 3. Because there were no known genealogical links between these pedigrees, the pSGS statistic in these two-pedigree analyses could not be weighted by the number of meioses between cases, so an un-weighted paired average method was used. Table 3 illustrates the results of these two-pedigree analyses. All regions remained at least nominally significant indicating that the underlying risk variants could be the same in the two pedigrees. Of particular note was the 7.6 Mb region on chromosome 4 that increased in significance, despite the potential loss of power due to our inability to weight the sharing by meioses in the two-pedigree analysis.

**Table 3:** pSGS results for two-pedigree analyses including pedigrees 1&3 in the overlapping regions

**Chr**	**Region**	**Length (Mb)**	**pSGS pedigree 1**	**pSGS pedigree 3**	**Two-ped-pSGS (local controls)**	**Av. sharers in 2-ped analysis (range)**
4	47,003,076 - 54,575,432	7.6	0.0060	0.013	0.0025	14.14 (10–15)
7	16,704,212 - 31,213,647	14.5	0.0050	0.0065	0.0076	14.00 (9–15)
14	66,272,834 - 77,581,481	11.3	0.040	0.0070	0.011	14.04 (9–15)

## Discussion

We investigated three extended Utah high-risk breast cancer pedigrees using weighted pSGS analysis to identify regions of excessive sharing that could potentially harbor breast cancer susceptibility loci. Five regions of interest were identified on chromosomes 2, 4, 7, 8 and 14. Three of these regions (chromosomes 4, 7 and 14) showed evidence for excessive sharing in two pedigrees (pedigrees 1 and 3), with chromosome 4 being perhaps of particular interest because the region gained significance in the two-pedigree analysis. All five of these regions have either been identified previously in genomewide searches or candidate susceptibility genes reside in them. Our region on chromosome 4 is supported by evidence from two previous genomewide linkage studies of families not attributable to *BRCA1* or *BRCA2*. A large international multi-center linkage study of 149 breast cancer families identified the chromosome 4 region as the best linkage across the genome (LOD=1.8) [19]. This location on chromosome 4 was also reported as one of the top candidate regions in another genomewide linkage scan (LOD=1.3) [20]. In addition, our region includes cytogenetic band 4q12 which has previously been proposed as a location potentially harboring genes important in breast cancer development because of observed loss of heterozygosity at 4q12 in both *BRCA1/2* and sporadic breast cancer tumors [21,22]. Furthermore, there has been recent interest in two candidate genes in this region, with increased gene copy number for genes *KIT* and *VEGFR2* found in triple negative breast cancer, an aggressive and difficult to treat form of the disease [23]. Our region on chromosome 14q includes the breast cancer candidate gene *RAD51L1*, which contains one of the two most significant associations reported in a multi-stage genomewide association study of 9,770 cases and 10,799 controls [24]. Our region on chromosome 7 contains the *AHR* gene that has been associated with breast cancer risk [25,26], and *IL6*[27], which contains a marker associated with increased risk for breast carcinoma [28]. Our 88.2 Mb region on chromosome 2 includes the gene *ZEB2* that is involved in RAS pathway that has been proposed as involved in clinical breast cancer progression [29]. There are also two SNPs (rs17188434 and rs12472911) that have been associated with age at menarche in this region [30], and early menarche is suggested to be a risk factor for breast cancer. The large 83.3 Mb region on chromosome 8 encompasses multiple possible breast cancer candidate genes: for example, *POLB*[31] and *NBS1* (NBN) [32,33] have previously been implicated in heritable susceptibility to breast cancer; *EBAG9* has been suggested to be involved in early stage breast cancer [34].

Seven nominal regions were also identified in our analyses. Notably two of these regions are on chromosome 17. One of the chromosome 17 regions in pedigree 1 (10.8-19.9 Mb) contains the candidate gene, *ELAC2*, which was previously proposed as a susceptibility gene for prostate cancer using Utah high-risk pedigrees [35]. Genes that increase susceptibility to both breast and prostate cancer have been observed previously; for example, *BRCA2*[36]. The second chromosome 17 region was found in pedigree 3 (32.8-51.1 Mb) and contains the high-risk breast cancer gene, *BRCA1*. It is perhaps surprising that a region containing *BRCA1* would arise, given our aim to screen out families with known BRCA mutations. In agreement with our selection criteria, we show no linkage at this locus (LOD=0.03). Nonetheless, it is possible that *BRCA1* remains a potential factor for risk in this pedigree.

We selected weighted pairwise SGS as our primary analysis specifically because the original SGS method will lose power quickly with intra-familial heterogeneity, and breast cancer is known to be a complex and very heterogeneous disease. In line with this assumption, only one of our genomewide suggestive pSGS regions also showed genomewide suggestive evidence using the original SGS algorithm. Furthermore, while the number of sharers across our regions of interest remained high, the range was wide and often reduced to the minimum possible number sharing. Hence, it appears that the pairwise algorithm may have been successful at providing more robustness to noise from heterogeneity, in addition to any residual genotyping error. One of our most significant pSGS regions (chromosomes 2) also showed genomewide suggestive evidence using multipoint linkage analysis with a dominant model.

An advantage of a pedigree design for gene identification is that a small number of cases and a well-delimited region can be easily defined and increases the efficiency of downstream experiments. Sequencing multiple cases selected for their high likelihood of sharing the underlying susceptibility variant provides an additional and powerful filter that can be used to parse findings from sequencing efforts. Hence, follow-up regionally-focused sequencing of the most compelling of these regions is a cost-effective and logical next step to identify the critical underlying risk variant at these loci.

## Conclusions

Our pSGS analyses have highlighted several regions that have the potential to harbor susceptibility variants for breast cancer, some of which confirm loci previously proposed by others. Three of our most significant regions (chromosomes 4, 7 and 14) were observed in two pedigrees and show evidence for shared risk variants across those pedigrees. Arguably, these three regions in pedigrees 1 and 3 are particularly good candidates to pursue using regionally-focused sequencing to identify novel breast cancer risk variants. In addition, and more broadly, this study has illustrated the potential utility of our new weighted pSGS method and extended pedigrees for gene mapping in complex diseases.

## Abbreviations

IBD: Identity-By-Descent; IBS: Identity-By-State; iControlDB: Illumina Genotype Control Database; LD: Linkage Disequilibrium; MAF: Minor Allele Frequency; MCMC: Markov chain Monte Carlo; pSGS: pairwise Shared Genomic Segment; SGS: Shared Genomic Segment; SNP: Single Nucleotide Polymorphism; UCR: Utah Cancer Registry; UPDB: Utah Population Database.

## Competing interests

The authors declare that they have no competing interests.

## Authors’ contributions

ZC performed the statistical analyses, drafted the manuscript and participated in algorithm and parallelization software development. AT participated in algorithm development, study design and statistical oversight. CT performed statistical analyses. JF contributed to data coordination and processing. LACA conceived of the study. NJC had oversight for study design, algorithm development, statistical analyses and manuscript writing. All authors read and approved the final manuscript.
